# Accelerometer-determined physical activity and cognitive function in middle-aged and older adults from two generations of the Framingham Heart Study

**DOI:** 10.1016/j.trci.2019.08.007

**Published:** 2019-10-15

**Authors:** Nicole L. Spartano, Serkalem Demissie, Jayandra J. Himali, Kimberly A. Dukes, Joanne M. Murabito, Ramachandran S. Vasan, Alexa S. Beiser, Sudha Seshadri

**Affiliations:** a Section of Endocrinology, Diabetes, Nutrition and Weight Management, Boston University School of Medicine, Boston, MA, USA; b Framingham Heart Study, Framingham, MA, USA; c Department of Biostatistics, Boston University School of Public Health, Boston, MA, USA; d Department of Neurology, Boston University School of Medicine, Boston, MA, USA; e Biostatistics and Epidemiology Data Analysis Center, Boston University School of Public Health, Boston, MA, USA; f Section of General Medicine, Boston University School of Medicine, Boston, MA, USA; g Section of Preventive Medicine and Epidemiology, Section of Cardiovascular Medicine, Department of Medicine, Boston University School of Medicine, Boston, MA, USA; h Glenn Biggs Institute for Alzheimer's and Neurodegenerative Diseases, University of Texas Health Sciences Center, San Antonio, TX, USA

**Keywords:** Cognition, Executive function, Memory, Physical activity, Exercise, Accelerometer, Moderate-to-vigorous, Sedentary time, Epidemiology

## Abstract

**Introduction:**

Physical activity (PA) may play a role in maintenance of cognitive function in both middle and older ages and prevention of outcomes such as dementia and Alzheimer's disease.

**Methods:**

Cross-sectional regression analyses were performed in Framingham Heart Study Third Generation (n = 1861) and Offspring (n = 909) cohort participants assessing the association of accelerometry-measured PA with cognitive function, adjusting for age, sex, accelerometer wear time, education, occupational status/PA, and smoking status.

**Results:**

In each cohort, achieving just 10–21.4 min/day moderate-to-vigorous PA related to better executive function (*P* < .02); and just 10 min/day moderate-to-vigorous PA was associated with better verbal memory in middle-aged adults in the Third Generation cohort (*P* = .02). In older adults of the Offspring cohort, total PA (measured in steps/day) was associated with better executive function (*P* < .02).

**Discussion:**

PA at levels lower than the current PA Guidelines (just 10 min/day moderate-to-vigorous PA and total PA including lower intensity PA) were associated with better cognitive function.

## Highlights

• Physical activity related to better cognition in middle-aged and older adults.
• Just 10 minutes of moderate-to-vigorous activity related to better cognition.
• Walking more total steps/day is related to better executive function in older adults.

## 1 Introduction

There are currently limited treatment options for dementia and Alzheimer's disease (AD). Therefore, prevention efforts, such as increasing physical activity (PA), may be one of the most promising strategies in combating these diseases [1]. Currently, few adults, especially older adults, meet the PA Guidelines for Americans, set by the US Health and Human Services of at least 150 minutes of moderate-to-vigorous (MV) PA per week [2], [3]. It is unclear whether these PA levels are feasible for older adults or whether these doses are appropriate for the prevention of cognitive decline or dementia/AD [4]. The most recent update to the PA Guidelines, released in 2018, suggests that older adults who are unable to achieve these recommendations can still obtain health benefits from participating in a lower, more tolerable PA level [2]. But there is a need for more research designed to reveal the precise doses (duration, intensity, and frequency) of PA associated with maintaining cognitive function into older adulthood [5].

A number of longitudinal cohort studies have observed associations of low self-reported PA with cognitive decline, dementia, and AD [1], [6], [7], [8], [9]. These studies used subjective PA methods that may introduce misclassification and obscure the true dose of PA that relates most favorably to cognition. The ability to measure precise doses of PA and sedentary time (SED) objectively, using accelerometers, provides an opportunity to clearly define the role of PA and SED in cognitive function in older adults. Thus far, studies investigating cross-sectional associations between objectively measured PA methods and cognitive function have led to inconsistent findings [10], [11] due to a host of factors including demographic differences in study populations, including age differences. In the current cross-sectional investigation, we explored associations of objectively measured PA and SED with cognitive function in two different generations of the Framingham Heart Study (FHS), the older Offspring cohort and the Third Generation cohort, consisting mainly of the middle-aged children of the Offspring cohort. Leveraging these two cohorts enables investigation of precise doses of PA and SED and of the influence of age, education, and occupational PA/status on the association between PA and SED with cognitive function.

## 2 Methods

### 2.1 Study population

In 1971, the children and the children's spouses of the original FHS cohort were recruited for the Framingham Offspring Study [12]. In 2002, children of the Offspring cohort were enrolled into the Third Generation cohort and new offspring spouses were also enrolled in 2002 and have been included in the Third Generation cohort in the current investigation [13]. We assessed cognitive function and PA in participants from FHS Third Generation (examination cycle 2, 2008–2011) and Offspring (examination cycle 9, 2011–2014) cohorts [13], [14]. From a total of 5841 who attended these examinations, 3765 completed the neuropsychological examinations, from which we excluded 186 participants who had dementia or stroke. From the remaining 3579 participants, we further excluded 809 participants who did not complete the PA study with at least 3 “valid” days (>10 hours/day), leaving a total sample of 2770 participants.

### 2.2 PA assessment

During Offspring examination 9 and Third Generation examination 2, all participants were asked to wear an omnidirectional Actical accelerometer (198-0200-00; Philips Respironics, Murrysville, PA, USA) for 8 days, attached to a belt worn around the waist. Third Generation participants were asked to wear the device for 24 hours each day and to only remove it when swimming and bathing; Offspring participants were asked to wear the device only during waking hours and to remove it for sleep, swimming, and bathing.

The Actical accelerometer records signals (0.5–3 Hz) and accelerations/decelerations (0.05–2 g) that are collected at 30-second intervals and grouped into “counts” or “steps.” For analysis, we used customized software (Kinesoft, version 3.3.63, Saskatchewan, Canada) to perform quality control, average signals over 1 minute, and define “valid” accelerometer data (if the device was worn for ≥10 hours/day for at least 3 days, not including the first day of wear, which was excluded from data processing). Nonwear time was defined as 60 consecutive minutes of zero counts, allowing for 2-minute interruption periods, and was removed from data processing. SED was defined as <200 counts/minute and was reported as % of wear time, only during the hours of 6 AM–10 PM and then standardized to a 16-hour day for those that did not wear the device for the full 16 hours. SED was presented as a continuous variable. These analytical decisions were carried out in both cohorts to minimize the effect of different amounts of wear time between cohorts. MVPA (>1486 counts/min) [15], [16] and the total number of steps per day were accumulated any time the device was worn (not restricted to 6 AM–10 PM) and presented categorically.

Accelerometer-determined PA is only modestly associated with self-reported PA in our study sample, using the FHS PA index (Spearman correlation coefficient for the association of self-reported PA with accelerometer-determined steps/day in our cohorts were 0.23 and 0.27, *P* < .0001 in Offspring and Third Generation, respectively, data not shown in tables).

### 2.3 Neuropsychological tests

Trained interviewers administered a battery of neurocognitive tests at Offspring examination 9 and Third Generation examination 2, using standard protocols [17]. The primary focus of this investigation was on executive function and verbal memory outcome measures. *Executive function* was assessed using Trail Making Test Part B minus Part A (Trails B − A); for this assessment, Trails B − A was transformed to normalize distribution by taking the log of (2-[Trails B − A]) and then re-signed, so that higher scores corresponded to better performance on these tasks. *Verbal memory* was assessed using the Logical Memory delayed recall component from the Wechsler Memory Scale. Additional tests assessing visual memory, abstract reasoning, and global cognition were also included in secondary analysis, described in the Supplementary Methods.

### 2.4 Covariates

The following covariates and cardiovascular risk factors were assessed: age, sex, education, retirement status and occupational PA/status (coded into 4 PA/occupation categories: retired/underemployed, sedentary occupation, light PA occupation, and moderate PA occupation), current smoking status, body mass index (BMI), diabetes mellitus (DM), hypertension (HTN), and cardiovascular disease (CVD). For the occupational status and occupational PA variables, participants reported whether they were employed full time, part-time, unemployed, or retired. Occupation data collected from participants were linked to Standard Occupation Codes from the US Census [18]. For participants working full time, we used published estimates of PA (metabolic equivalent) for each occupation [19], [20].

### 2.5 Statistical analysis

We present descriptive data for each cohort using means and standard deviations for continuous variables and frequency and percent for categorical variables. PA was assessed using steps per day (four categories), MVPA min/day (four categories), and SED min/day (continuous). Cognitive function was primarily assessed using executive function and verbal memory. Owing to differences in accelerometer wear time between cohorts (24 hours/day compared with only during waking hours), we decided, *a priori*, to present analyses for each cohort separately.

We tested associations between PA and constructs of cognitive function scores using multivariable linear and logistic regression, adjusting for age, sex, education, occupational PA/status, current smoking status, accelerometer wear time, and time between PA and cognitive assessments. In addition, to assess which PA component was the more predominant factor in relation to cognitive function, both MVPA and SED were analyzed simultaneously (in the same model). Despite moderate correlation between MVPA and SED (Spearman correlation coefficient in the Offspring cohort: −0.70, *P* < .0001; and in the Third Generation: −0.59, *P* < .0001), there is no indication for collinearity in our models (variance inflation factors <2 for all variables). For presentation in figures, dependent variables were standardized to a 0 mean and 1 standard deviation of the total population (including both Third Generation and Offspring) for least-squared means analysis, adjusting for age, sex, accelerometer wear time, education, occupational status/PA, smoking status, and time between PA and cognitive assessments (with MVPA models additionally adjusted for SED). We also evaluated models that adjusted for additional cardiovascular risk factors (BMI, DM, HTN, and prevalent CVD) and assessed potential selection bias using the χ^2^ analysis and t-tests to compare demographic details from participants who did and did not complete the PA accelerometry study. Association estimates from regression models were considered significant at *P* ≤ .05 level. Analyses were performed using the Statistical Analyses System software, version 9.4 (SAS Institute, Cary, NC).

## 3 Results

### 3.1 Cohort demographic differences

The Framingham Third Generation participants were younger than the Offspring participants (mean age 48.7 ± 8.6 vs. 71.3 ± 7.6 years, Table 1), were more likely to have completed college education (57% vs. 47%), had higher cognitive function scores, and were less often retired or underemployed (26% vs. 76%); almost 55% of Offspring participants were retired at the time of the examination. Finally, as expected for an older cohort, the Offspring participants were less physically active than the Third Generation cohort, having almost 10 fewer minutes of MVPA per day and 1725 fewer steps, on average. Only 31% of the Offspring cohort, compared with 53% of the Third Generation cohort, met the current PA Guidelines (150 minutes/week or 21.4 minutes/day of MVPA).

**Table 1:** Descriptive data

Study characteristics	Third Generation (n = 1861)	Offspring (n = 909)
Age, years	48.7 ± 8.6	71.3 ± 7.6
Men, n (%)	865 (47)	404 (44)
BMI, kg/m^2^	27.8 ± 5.5	27.7 ± 4.8
Smoking status, n (%)	148 (8)	41 (5)
Diabetes mellitus, n (%)	82 (4)	112 (13)
Hypertension, n (%)	441 (24)	506 (56)
CVD, n (%)	37 (2)	107 (12)
Education, n (%)		
No HS degree	7 (<1)	12 (1)
HS degree	240 (13)	199 (22)
Some college	563 (30)	267 (29)
College graduate	1051 (57)	431 (47)
Retirement status, n (%)	69 (3.7)	491 (54.6)
Current Occupational PA, n (%)		
Retired or underemployed	486 (26)	685 (76)
Sedentary occupation	627 (34)	92 (10)
Light occupation	635 (34)	111 (12)
Moderate occupation	105 (6)	12 (1)
Cognitive function		
Trails B − A∗mean (IQR)	0.61 (0.38, 0.72)	1.07 (0.52, 1.15)
Learning Memory–delayed	11.6 ± 3.7	11.1 ± 3.8
Objective physical activity data		
Total steps/day, mean ± SD	8526 ± 3836	6801 ± 3936
Total steps/day by category, n (%)	288 (16)	327 (36)
<5k/day		
5–7.5k/day	557 (30)	243 (27)
7.5–10k/day	491 (26)	179 (20)
10k+/day	525 (28)	160 (18)
MVPA min/day, mean ± SD	27.7 ± 20.9	18.8 ± 25.7
MVPA by category, n (%)		
<10 min/day (<70 min/week)	333 (18)	417 (46)
10–21.4 min/day (70–150 min/week)	541 (29)	312 (23)
21.4–30 min/day (150–210 min/week)†	310 (17)	98 (11)
30+ min/day (≥210 min/week)	677 (36)	181 (20)
SED min/day‡ [standardized to 6 AM–10 PM day], mean ± SD	781 ± 59	810 ± 65
Accelerometer wear time (6 AM–10 PM), mean ± SD	817 ± 77	747 ± 72

NOTE. Underemployed individuals are defined as retired, unemployed, or working part time.

Abbreviations: MVPA, moderate-to-vigorous physical activity; SED, sedentary time; SD, standard deviation; CVD, cardiovascular disease; BMI, body mass index; IQR, interquartile range.

∗ Trails B − A was transformed for analysis by taking the log of (2-[Trails B − A]) and then re-signed, so that higher scores corresponded to better performance on these tasks.

† 150 min/week MVPA is consistent with meeting the PA Guidelines.

‡ SED is reported as a percent of wear time and then standardized to a 16-hour day.

### 3.2 Relations of PA to executive function

**Fig. 1:**
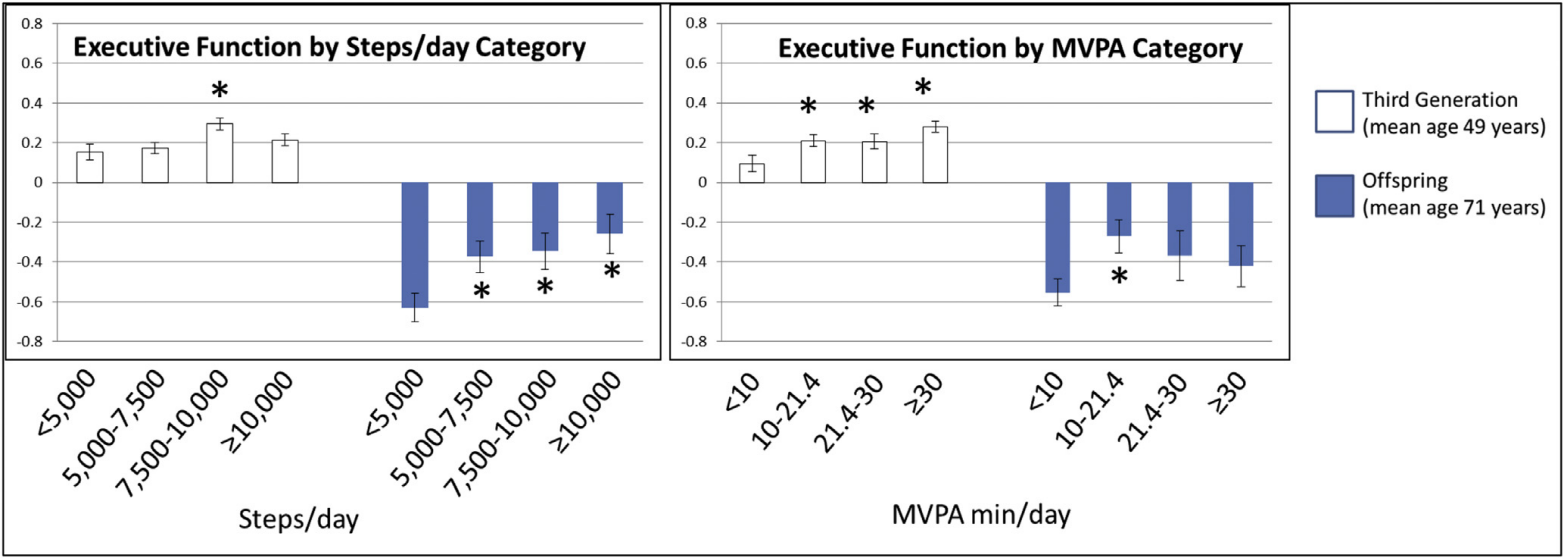
Least squared means of executive function measures by physical activity categories. ∗ Indicates a significant difference from the reference categories (<10 min/day MVPA or <5000 steps/day) within each cohort (Third Generation or Offspring). Executive function (Trails B − A) is standardized to the mean and standard deviation of the total study population. Adjustment model includes age, sex, accelerometer wear time, education, occupational status/PA, smoking status, and time between PA and cognitive assessments. MVPA models were additionally adjusted for SED. Abbreviations: MVPA, moderate-to-vigorous physical activity; SED, sedentary time.

Achieving an average of 10–21.4 minutes of MVPA/day was associated with better *executive function* in both middle aged (Third Generation cohort, β = 0.02 ± 0.009, *P* = .02, Table 2 and Fig. 1) and older adults (Offspring cohort, β = 0.06 ± 0.02, *P* = .01) compared with achieving <10 minutes of MVPA/day, even after adjusting for covariates including SED. We also observed a dose-response association, in which higher total PA (measured in steps/day) associated with better *executive function* in the older Offspring cohort (β = 0.05–0.07 ± 0.02, *P* < .02 for achieving increments greater than 5000 steps/day). Analysis presented in Fig. 1, in which executive function was standardized to the mean and standard deviation of the total study population (including Third Generation and Offspring), demonstrates differences in executive function by cohort and by PA. SED was not significantly associated with *executive function* in either cohort after adjusting for covariates including MVPA.

**Table 2:** The associations of physical activity and sedentary time with **executive function** (as assessed by Trails B − A)

	Categories or continuous	Model	Third Generation (n = 1808)		Offspring (n = 866)	
			β ± SE	*P*	β ± SE	*P*
Steps/day	<5k	1	Ref		Ref	
	5–7.5k		0.004 ± 0.009	.69	0.05 ± 0.02	.02
	7.5–10k		0.03 ± 0.01	.006	0.06 ± 0.02	.02
	10k+		0.01 ± 0.01	.23	0.07 ± 0.02	.004
MVPA, min/d∗	<10	1+SED	Ref		Ref	
	10–21.4†		0.02 ± 0.009	.02	0.05 ± 0.02	.01
	21.4–30		0.02 ± 0.01	.05	0.04 ± 0.03	.22
	30+		0.04 ± 0.01	.001	0.03 ± 0.03	.33
SED∗	(per 1 h/day)	1+MVPA	0.005 ± 0.004	.177	−0.02 ± 0.01	.07

NOTE. Adjustment model 1: age, sex, accelerometer wear time, education, occupational status/PA, smoking status, and time between PA and cognitive assessments.

Abbreviations: MVPA, moderate-to-vigorous physical activity; SED, sedentary time; SE, standard error.

∗ MVPA models were additionally adjusted for SED; and SED models were additionally adjusted for MVPA.

† 21.4 min/day MVPA cutoff represents 150 min/week MVPA, consistent with recommendations in the Physical Activity Guidelines for Americans.

### 3.3 Relations of PA to verbal memory and global cognition

**Fig. 2:**
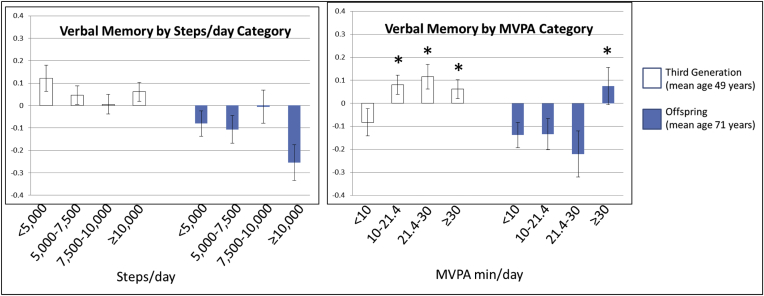
Least squared means of verbal memory measures by physical activity categories. ∗ Indicates a significant difference from the reference categories (<10 min/day MVPA or <5000 steps/day). Verbal memory (Learning Memory–delayed task) is standardized to the mean and standard deviation of the total study population. Adjustment model includes age, sex, accelerometer wear time, education, occupational status/PA, smoking status, and time between PA and cognitive assessments. MVPA models were additionally adjusted for SED. Abbreviations: MVPA, moderate-to-vigorous physical activity; SED, sedentary time.

In the middle-aged, Third Generation cohort, we observed a positive dose-response association of MVPA with *verbal memory* (Table 3 and Fig. 2) and *global cognitive score* (Supplementary Table 2), after adjusting for covariates including SED; achieving 10 min/day MVPA was associated with better *verbal memory* (β = 0.61 ± 0.26, *P* = .02) compared with the referent level (<10 min/day); and meeting the PA Guidelines (150 min/week [21.4 min/day] MVPA) was associated with even greater *verbal memory* (β = 0.74 ± 0.30, *P* = .02). In the older, Offspring cohort, only achieving ≥30 min/day MVPA had a borderline association with better verbal memory, compared with achieving <10 min/day (β = 0.80 ± 0.40, *P* = .05). Unexpectedly, SED was positively related to *verbal memory*, *global cognitive score*, and *visual reproduction/memory*, even after adjusting for MVPA in the Third Generation cohort (β = 0.24 ± 0.10, *P* = .01; β = 0.07 ± 0.02, *P* = .002, β = 0.20 ± 0.07, *P* = .004, Table 3 and Supplementary Methods and Supplementary Tables 2 and 3). PA variables were not significantly associated with *visuoperceptual skills* or *abstract reasoning*, as assessed using Hooper Visual Organization and Similarities tests, respectively (*P* > .05, Supplementary Methods and Supplementary Tables 4 and 5). After adjustment for cardiometabolic risk factors (BMI, DM, HTN, and CVD), data parameter estimates and level of significance did not substantially change for any associations measured, data not shown.

**Table 3:** The associations of physical activity and sedentary time with **verbal memory** (as assessed by the Learning Memory–delayed task)

	Categories or continuous	Model	Third Generation (n = 1845)		Offspring (n = 886)	
			β ± SE	*P*	β ± SE	*P*
Steps/day	<5k	1	Ref		Ref	
	5–7.5k		−0.28 ± 0.26	.28	−0.10 ± 0.32	.76
	7.5–10k		−0.435 ± 0.27	.11	0.28 ± 0.35	.43
	10k+		−0.23 ± 0.27	.40	−0.65 ± 0.38	.08
MVPA, min/d∗	<10	1+SED	Ref		Ref	
	10–21.4†		0.61 ± 0.26	.02	0.01 ± 0.34	.97
	21.4–30		0.74 ± 0.30	.02	−0.31 ± 0.45	.49
	30+		0.54 ± 0.29	.06	0.80 ± 0.40	.05
SED∗	(per 1 h/day)	1+MVPA	0.24 ± 0.10	.01	0.26 ± 0.15	.08

NOTE. Adjustment model 1: age, sex, accelerometer wear time, education, occupational status/PA, smoking status, and time between PA and cognitive assessments.

Abbreviations: MVPA, moderate-to-vigorous physical activity; SED, sedentary time; SE, standard error.

∗ MVPA models were additionally adjusted for SED; and SED models were additionally adjusted for MVPA.

† 21.4 min/day MVPA cutoff represents 150 min/week MVPA, consistent with recommendations in the Physical Activity Guidelines for Americans.

### 3.4 Demographics and cognitive function of excluded participants

We also reported demographics for participants who did not complete the PA-accelerometry assessment in order to understand potential selection bias (Supplementary Table 1). In both cohorts, individuals with higher cognitive scores were more likely to participate in the PA-accelerometry study (for all cognitive scores: Offspring, *P* < .0001; Third Generation, *P* < .05). Offspring that participated in PA-accelerometry were also younger, with better general health (age 71 vs. 74 years, and with lower BMI, DM, HTN, and CVD, all *P* < .001) than those that did not participate. Third Generation PA accelerometry participants did not have significantly different ages but did have lower BMI and were less likely to smoke or have DM than those who did not participate in the PA accelerometry study (all *P* < .05).

## 4 Discussion

In subsamples from two cohorts of the community-based FHS, we observed statistically significant associations of greater PA with higher executive function. In the younger of these two cohorts (the middle-aged Third Generation cohort), associations were mostly limited to that of MVPA with executive function; but in the older Offspring cohort, we observed that a higher total volume of PA (measured by total step accumulation) was also associated with better executive function. In the Third Generation cohort, MVPA was also related to better performance on the verbal memory tasks and to high scores on the global cognitive function composite. Finally, we observed an unexpected result in the Third Generation cohort, in which SED was positively associated with higher verbal memory and global cognitive score. Our findings show some similarities with previous epidemiological investigations but also provide novel insights which will require further examination.

In agreement with our results in the FHS cohorts (including mostly white individuals of European descent), previous studies also conducted mostly in white, non-Hispanic/Latino populations have consistently reported associations of objectively measured PA with executive function; but these results have not been consistently observed in studies conducted in black or Hispanic/Latino adults [10], [11], [21], [22]. Possible explanations of differing results among these studies include methodological and demographic differences other than race/ethnicity, including age, education, geographic region, socioeconomic status, and method of defining PA variables, such as differing intensity threshold for cutpoints or whether the variable was indexed to wear time.

Two major studies contributing to our understanding of cross-sectional relations of objectively measured PA to cognitive function include the Reasons for Geographic and Racial Differences in Stroke (REGARDS) study of black and white adults ≥60 years and the Hispanic Community Health Study/Study of Latinos (HCHS/SOL) of mostly middle-aged Hispanic/Latino adults [10], [11]. In a subanalysis of only white participants in REGARDS, an association was observed between %MVPA (reported as % of total device wear time) and executive function, which was not observed in black participants [10]. But in the full REGARDS study sample (including both black and white adults) and HCHS/SOL, there were more inconsistent results [10], [11]. In REGARDS, %MVPA was associated with a z-score composite of multiple cognitive assessments in the full sample and with memory in both black and white men; whereas in the mostly white Framingham cohorts, we observed similar associations only in our middle-aged Third Generation cohort, but not in the older Offspring cohort. Using the more traditional MVPA (min/day) definition (adjusting for wear time), no statistically significant association was observed with a global cognitive function score in the HCHS/SOL cohort [11]. REGARDS and HCHS/SOL also defined MVPA using different thresholds (≥1065 vs. ≥1535, compared with the threshold used in our study ≥1487 counts/min). However, one consistency among REGARDS, HCHS/SOL, and FHS are that we all used the same type of device to measure PA (Actical; Philips Respironics). Future projects are underway to harmonize PA data analysis across cohorts to identify potential reasons for the different results observed in our cohorts.

In agreement with our cross-sectional data, there have been a few prospective studies indicating that higher objectively-measured PA and fitness are associated with less cognitive decline, impairment, and dementia/AD [23], [24], [25], [26]. There is also a breadth of data suggesting that self-reported PA is associated with better cognitive outcomes [1], [6], [7], [8], [9]. But randomized controlled trials of exercise interventions have been more inconsistent, possibly due to population differences, exercise protocol differences, and necessarily short follow-up times of lifestyle interventions [4], including a recent clinical trial reporting that participation in an MVPA intervention resulted in steeper cognitive decline in patients with dementia [27]. In a striking positive example, Erickson et al. [28] reported a better maintenance of hippocampal volume (measured by magnetic resonance imaging) and memory in older adults without baseline cognitive impairment who were exposed to a one-year exercise intervention, compared with the control group. However, the most consistent findings across randomized controlled trial exercise interventions are those linking more exercise to better executive function, attention, and processing speed [29], [30], [31]. Similarly, our observed association of PA with executive function was the most consistent result across both cohorts.

Despite some consistency in observing associations of PA with executive function, the diversity of other favorable cognitive outcomes associated with PA in different studies suggests mechanisms influencing many cognitive domains [1], [6], [7], [8], [9], [23], [24], [25], [26], [28], [29], [30], [31]. Dementia and AD share many of the same risk factors as CVD, such as DM, obesity, HTN, high cholesterol levels, and smoking [5]; therefore, it has been hypothesized that much of the effect of PA on brain health may be mediated by cardiometabolic factors. In secondary analysis, we explored models adjusting for cardiometabolic risk factors and observed that these adjustments did not substantially impact the parameter estimates in analyses relating PA measures to cognitive function. However, vascular/cardiometabolic factors may still play a role in the associations we observed. The scientific report submitted by the PA Guidelines Committee in 2018 suggests that more long-term randomized controlled trials are needed to develop appropriate guidelines for brain health and also to understand how different biomarkers (e.g., circulating neurotrophic factors and brain imaging markers) influence the response to exercise or PA [4].

### 4.1 Discussing our unexpected findings: more sedentary time relates to better cognitive function in middle-aged adults

In the middle-aged Third Generation cohort, we observed a positive association of SED with verbal memory and global cognitive function. We theorized that the high SED associated with a cognitively complex occupation may account for some of the association of higher SED with higher global cognition. We adjusted for occupational status and occupational PA, but we only had crude occupational categorizations available, which may require follow-up analysis. In the HCHS/SOL study, investigators also observed a trend toward a positive association of SED with global cognitive function in middle aged adults (45–54 years), although this trend was not statistically significant [11]. Occupations with high cognitive complexity have previously been associated with higher cognitive function [32] and lower risk of dementia [33]; and many of these higher occupational complexity jobs also have lower occupational PA [33]. Future research is needed to identify whether occupations with high complexity also may have large amounts of sedentary time and computer use. Interestingly, computer use has been associated with higher cognitive function, whereas television-viewing was associated with the opposite effect in a large study of older French adults [34]. Similarly, other cognitively stimulating sedentary activities such as reading, playing games, or puzzles are associated with better cognitive performance [35], [36] as a component of the cognitive reserve model in preserving cognitive ability in older age [37].

### 4.2 Strengths and limitations

The availability of an objective measure of PA in two large cohorts within the FHS, with different mean ages, is a strength of this study. Most previous research studies in this area have used self-reported PA, which introduces substantial misclassification [38]. Accelerometers provide a more accurate method to sensitively test the dose (intensity and total volume) of PA associated with health outcomes, and as we reported, only modestly correlate with self-reported PA. In contrast, accelerometer determination of SED is a more challenging variable to assess because of difficulty in distinguishing SED from standing or from times when the device was not being worn (nonwear time), which is a limitation of this study. We are able to partially address bias introduced by differences in wear time among individuals by adjusting for wear time, but the influence of differential wear time especially on SED remains a limitation. We must also acknowledge selection bias in our sample. Participants who either chose not to participate in our accelerometry study or provided incomplete data were older, less educated, and had lower cognitive function. It is unclear how inclusion of these individuals in our sample may have impacted our results. Owing to the cross-sectional nature of our investigation, it is also not possible to determine causation and a bidirectional relationship is likely.

Another consideration for accelerometry studies is that absolute levels of PA should be translated cautiously. Other studies have used different devices (with inherent differences in units, step algorithms, and sensitivity to low frequency [slow] movement), intensity cutpoints, and data censoring methods [39], [40], [41]. Although other investigators are exploring the use of lower thresholds for MVPA in older adults to account for potential differences in fitness or functional ability [42], we chose to use one standard threshold for all participants, so we could compare the effect of similar doses of PA on our different cohorts.

The association we observed of total PA (measured in steps/day) with executive function in older adults is novel because there have been no other accelerometer studies that have related steps to cognitive function in our review of the published literature. These results could be explored in future analysis to better understand the dose of activity necessary to achieve benefit in older adults.

## 5 Conclusions

Incrementally higher doses (duration, intensity and frequency) of PA, at levels lower than the PA Guidelines for Americans, were related to better executive function in middle-aged and older adults. Future studies are necessary to explore the unexpected association of higher SED with higher global cognition, which may be due to unmeasured factors such as the cognitive complexity of sedentary occupations in middle age. Finally, similar to other converging information, our study offers further evidence that total PA, not just MVPA, may be important for maintaining cognition in older adults.

**Research in Context**

1. Systematic review: Evidence from previous literature suggests that physical activity (PA) may be related to cognitive function, but results have been inconsistent, partially due to subjective methods of assessing PA and demographic differences in study populations.
2. Interpretation: Our results, using objective PA assessment methods, indicate that both moderate-to-vigorous and total PA (which also includes activities of lower intensity) are related to better maintenance of executive function in older adults; whereas only moderate-to-vigorous intensity PA was related to better cognitive function in middle-aged adults.
3 Future directions: Next steps for this research are to replicate our results in other cohorts and test our hypotheses in intervention studies, investigating associations between total PA and cognitive function across race/ethnicities and socioeconomic groups.
