# Supplementary material for: Accelerometer-determined physical activity and cognitive function in middle-aged and older adults from two generations of the Framingham Heart Study
Source: Alzheimers Dement (N Y). 2019 Oct 15;5:618–26. doi: 10.1016/j.trci.2019.08.007 (PMC6807299; doi:10.1016/j.trci.2019.08.007)
Supplement: Supplementary Material [file mmc1.docx]

**Supplemental Methods:**

*Visual memory was* assessed using the Visual Reproduction-delayed memory task; *visuoperceptual skills* were assessed using Hooper Visual Organization; and *abstract reasoning* was assessed using Similarities tests. Hooper Visual Organization was natural log transformed to normalize its distribution. We also performed PC-1 analysis with weights modified from methods developed in a cross-cohort analysis of *global cognition score* using a subset of unrelated Framingham Heart Study participants (n=1889)[1] and tests that have been previously described:[2] Similarities=0.24, Ln-Trail Making Test B=0.25, Logical Memory (sum of immediate and delayed recall scores)=0.22, Visual Reproduction Memory (sum of immediate and delayed recall scores)=0.27, Paired Associate Learning (sum of immediate and delayed recall scores)=0.24, and Ln-Hooper Visual Organization test=0.24.

References

[1] Davies G, Lam M, Harris SE, Trampush J, Luciano M, Hill WD, et al. Ninety-nine independent genetic loci influencing general cognitive function include genes associated with brain health and structure (N = 280,360). bioRxiv. 2017.

[2] Au R, Seshadri S, Wolf PA, Elias M, Elias P, Sullivan L, et al. New norms for a new generation: cognitive performance in the framingham offspring cohort. Experimental aging research. 2004;30:333-58.

**Supplemental Table 1:** Cognitive function in participants without physical activity-accelerometer data

|  | Third Generation | | | Offspring | | |
| --- | --- | --- | --- | --- | --- | --- |
|  | Complete accelerometry data (n=1861) | Incomplete accelerometry data (n=413) | p-value for difference | Complete accelerometry data (n=909) | Incomplete accelerometry data (n=396) | p-value for difference |
| Age, years | 48.7 ± 8.6 | 48.2 ± 8.7 | 0.267 | 71.3 ± 7.6 | 74.1 ± 8.8 | **<0.0001** |
| Men, n (%) | 865 (46%) | 198 (48%) | 0.590 | 404 (44%) | 160 (40%) | 0.176 |
| BMI, kg/m^2^ | 27.8 ± 5.5 | 29.0 ± 6.7 | **0.0008** | 27.7 ± 4.7 | 29.7 ± 6.4 | **<0.0001** |
| Smoking status, n (%) | 148 (8%) | 60 (15%) | **<0.0001** | 41 (5%) | 22 (6%) | 0.417 |
| Diabetes mellitus, n (%) | 82 (4%) | 28 (7%) | **0.040** | 112 (13%) | 80 (22%) | **<0.0001** |
| Hypertension, n (%) | 441 (24%) | 102 (25%) | 0.687 | 506 (56%) | 287 (72%) | **<0.0001** |
| CVD, n (%) | 37 (2%) | 14 (3%) | 0.082 | 107 (12%) | 76 (19%) | **0.0004** |
| Education |  |  | 0.857 |  |  | **0.0001** |
| No HS degree | 7 (<1%) | 2 (<1%) |  | 12 (1%) | 17 (4%) |  |
| HS degree | 240 (13%) | 58 (14%) |  | 199 (22%) | 103 (26%) |  |
| Some college | 563 (30%) | 118 (29%) |  | 267 (29%) | 130 (33%) |  |
| College grad | 1051 (56%) | 235 (57%) |  | 431 (47%) | 146 (37%) |  |
| Retirement status | 69 (4%) | 15 (4%) | 0.929 | 491 (55%) | 259 (66%) | **0.0002** |
| Global Cognition (Principal Component-1) | 0.59 ± 0.81 | 0.38 ± 0.79 | **<0.0001** | -0.03 ± 0.99 | -0.52 ± 1.09 | **<0.0001** |
| Trails B-A | -0.95 ± 0.13 | -0.97 ± 0.16 | **0.005** | -1.08 ± 0.26 | -1.17 ± 0.32 | **<0.0001** |
| Learning Memory-delayed | 11.6 ± 3.7 | 11.1 ± 3.7 | **0.005** | 11.1 ± 3.8 | 10.0 ± 4.0 | **<0.0001** |
| Visual Reproductions-delayed | 9.0 ± 2.6 | 8.5 ± 2.6 | **0.0003** | 7.3 ± 3.0 | 5.8 ± 3.1 | **<0.0001** |
| Similarities | 17.4 ± 3.1 | 17.0 ± 3.1 | **0.014** | 17.2 ± 3.6 | 15.7 ± 3.7 | **<0.0001** |
| Hooper Visual Organization Task | -1.36 ± 0.51 | -1.48 ± 0.50 | **<0.0001** | -1.62 ± 0.49 | -1.79 ± 0.51 | **<0.0001** |

Abbreviations: Moderate to vigorous physical activity (MVPA); sedentary time (SED); standard error (SE).

Bolded p-values indicated statistical significance in comparing those with complete vs. incomplete accelerometry data within a given cohort, p<0.05

**Supplemental Table 2.** The associations of physical activity and sedentary time with **global cognitive score** (as assessed by a principal component analysis of multiple cognitive scores)

|  | Categories or continuous | Model | Third Generation (n=1698) | | Offspring (n=801) | |
| --- | --- | --- | --- | --- | --- | --- |
|  |  |  | β ± SE | p | β ± SE | p |
| Steps | <5k | 1 | ref |  | ref |  |
|  | 5-7.5k |  | 0.005 ± 0.057 | 0.925 | -0.007 ± 0.074 | 0.927 |
|  | 7.5-10k |  | -0.009 ± 0.059 | 0.878 | 0.015 ± 0.081 | 0.859 |
|  | 10k+ |  | -0.063 ± 0.059 | 0.290 | -0.098 ± 0.088 | 0.266 |
| MVPA, min/d† | <10 | 1+SED | ref |  | ref |  |
|  | 10-21.4 |  | **0.151 ± 0.056** | **0.007** | 0.040 ± 0.078 | 0.609 |
|  | 21.4-30 |  | 0.111 ± 0.065 | 0.086 | 0.024 ± 0.103 | 0.819 |
|  | 30+ |  | **0.149 ± 0.062** | **0.017** | 0.101 ± 0.093 | 0.277 |
| SED† | (min/day) | 1+MVPA | **0.069 ± 0.022** | **0.002** | 0.050 ± 0.034 | 0.136 |

Abbreviations: Moderate to vigorous physical activity (MVPA); sedentary time (SED); standard error (SE).

Adjustment model 1: age, sex, accelerometer wear time, education, occupational status/PA, smoking status, and time between PA and cognitive assessments. Bolded p-values indicated statistical significance, p<0.05.

†MVPA models were additionally adjusted for SED; and SED models were additionally adjusted for MVPA

**Supplemental Table 3.** The associations of physical activity and sedentary time with the visual reproduction-delayed task

|  | Categories or continuous | Model | Third Generation (n=1843) | | Offspring (n=888) | |
| --- | --- | --- | --- | --- | --- | --- |
|  |  |  | β ± SE | p | β ± SE | p |
| Steps | <5k | 1 | ref |  | ref |  |
|  | 5-7.5k |  | -0.102 ± 0.179 | 0.569 | 0.174 ± 0.236 | 0.462 |
|  | 7.5-10k |  | 0.056 ± 0.186 | 0.764 | -0.090 ± 0.263 | 0.732 |
|  | 10k+ |  | -0.350 ± 0.187 | 0.062 | -0.287 ± 0.281 | 0.307 |
| MVPA, min/d† | <10 | 1+SED | ref |  | ref |  |
|  | 10-21.4 |  | 0.337 ± 0.177 | 0.057 | 0.001 ± 0.253 | 0.996 |
|  | 21.4-30 |  | 0.093 ± 0.208 | 0.656 | -0.016 ± 0.333 | 0.962 |
|  | 30+ |  | 0.243 ± 0.200 | 0.220 | -0.047 ± 0.301 | 0.875 |
| SED† | (min/day) | 1+MVPA | **0.204 ± 0.070** | **0.004** | 0.115 ± 0.109 | 0.290 |

Abbreviations: Moderate to vigorous physical activity (MVPA); sedentary time (SED); standard error (SE).

Adjustment model 1: age, sex, accelerometer wear time, education, occupational status/PA, smoking status, and time between PA and cognitive assessments. Bolded p-values indicated statistical significance, p<0.05.

†MVPA models were additionally adjusted for SED; and SED models were additionally adjusted for MVPA

**Supplemental Table 4.** The associations of physical activity and sedentary time with the similarities task

|  | Categories or continuous | Model | Third Generation (n=1861) | | Offspring (n=909) | |
| --- | --- | --- | --- | --- | --- | --- |
|  |  |  | β ± SE | p | β ± SE | p |
| Steps | <5k | 1 | ref |  | ref |  |
|  | 5-7.5k |  | -0.120 ± 0.217 | 0.579 | -0.063 ± 0.281 | 0.822 |
|  | 7.5-10k |  | -0.296 ± 0.226 | 0.191 | -0.052 ± 0.312 | 0.868 |
|  | 10k+ |  | -0.131 ± 0.227 | 0.566 | -0.362 ± 0.334 | 0.279 |
| MVPA, min/d† | <10 | 1+SED | ref |  | ref |  |
|  | 10-21.4 |  | 0.268 ± 0.215 | 0.213 | -0.288 ± 0.300 | 0.337 |
|  | 21.4-30 |  | 0.286 ± 0.252 | 0.256 | -0.266 ± 0.396 | 0.502 |
|  | 30+ |  | 0.446 ± 0.243 | 0.066 | 0.431 ± 0.358 | 0.228 |
| SED† | (min/day) | 1+MVPA | 0.143 ± 0.086 | 0.095 | 0.104 ± 0.129 | 0.423 |

Abbreviations: Moderate to vigorous physical activity (MVPA); sedentary time (SED); standard error (SE).

Adjustment model 1: age, sex, accelerometer wear time, education, occupational status/PA, smoking status, and time between PA and cognitive assessments.

†MVPA models were additionally adjusted for SED; and SED models were additionally adjusted for MVPA

**Supplemental Table 5.** The associations of physical activity and sedentary time with the Hooper Visual Organization task

|  | Categories or continuous | Model | Third Generation (n=1836) | | Offspring (n=873) | |
| --- | --- | --- | --- | --- | --- | --- |
|  |  |  | β ± SE | p | β ± SE | p |
| Steps | <5k | 1 | ref |  | ref |  |
|  | 5-7.5k |  | 0.027 ± 0.037 | 0.454 | 0.017 ± 0.041 | 0.670 |
|  | 7.5-10k |  | -0.018 ± 0.038 | 0.642 | 0.027 ± 0.045 | 0.550 |
| MVPA, min/d† | <10 | 1+SED | ref |  | ref |  |
|  | 10-21.4 |  | 0.036 ± 0.036 | 0.320 | -0.030 ± 0.043 | 0.493 |
|  | 21.4-30 |  | -0.046 ± 0.043 | 0.276 | -0.032 ± 0.057 | 0.574 |
|  | 30+ |  | -0.010 ± 0.041 | 0.810 | -0.002 ± 0.052 | 0.967 |
| SED† | (min/day) | 1+MVPA | 0.003 ± 0.002 | 0.254 | 0.003 ± 0.003 | 0.404 |

Abbreviations: Moderate to vigorous physical activity (MVPA); sedentary time (SED); standard error (SE).

Adjustment model 1: age, sex, accelerometer wear time, education, occupational status/PA, smoking status, and time between PA and cognitive assessments. The outcome measure was log-transformed to normalize distribution.

†MVPA models were additionally adjusted for SED; and SED models were additionally adjusted for MVPA
